# Alzheimer’s disease: new insight in assessing of amyloid plaques morphologies using multifractal geometry based on Naive Bayes optimized by random forest algorithm

**DOI:** 10.1038/s41598-023-45972-w

**Published:** 2023-10-30

**Authors:** Elshaimaa Amin, Yasmina M. Elgammal, M. A. Zahran, Mohamed M. Abdelsalam

**Affiliations:** 1 Future Higher Institute of Engineering and Technology, Mansoura, Egypt; 2 Theoretical Physics Group, Physics Department, Faculty of Science, Mansoura University, Mansoura, Egypt; 3 Computers Engineering and Control Systems Department, Faculty of Engineering, Mansoura University, Mansoura, Egypt

**Keywords:** Biophysics, Engineering, Physics

## Abstract

Alzheimer’s disease (AD) is a physical illness, which damages a person’s brain; it is the most common cause of dementia. AD can be characterized by the formation of amyloid-beta (Aβ) deposits. They exhibit diverse morphologies that range from diffuse to dense-core plaques. Most of the histological images cannot be described precisely by traditional geometry or methods. Therefore, this study aims to employ multifractal geometry in assessing and classifying amyloid plaque morphologies. The classification process is based on extracting the most descriptive features related to the amyloid-beta (Aβ) deposits using the Naive Bayes classifier. To eliminate the less important features, the Random Forest algorithm has been used. The proposed methodology has achieved an accuracy of 99%, sensitivity of 100%, and specificity of 98.5%. This study employed a new dataset that had not been widely used before.

## Introduction

Alzheimer's disease (AD) is one of the most dreadful and generic classes of dementia that causes a progressive loss of memory and cognitive function, leading to poor quality of life. It accounts for almost 60–80% of dementia cases and it is ranked globally as the fifth leading cause of death.

**Figure 1:**
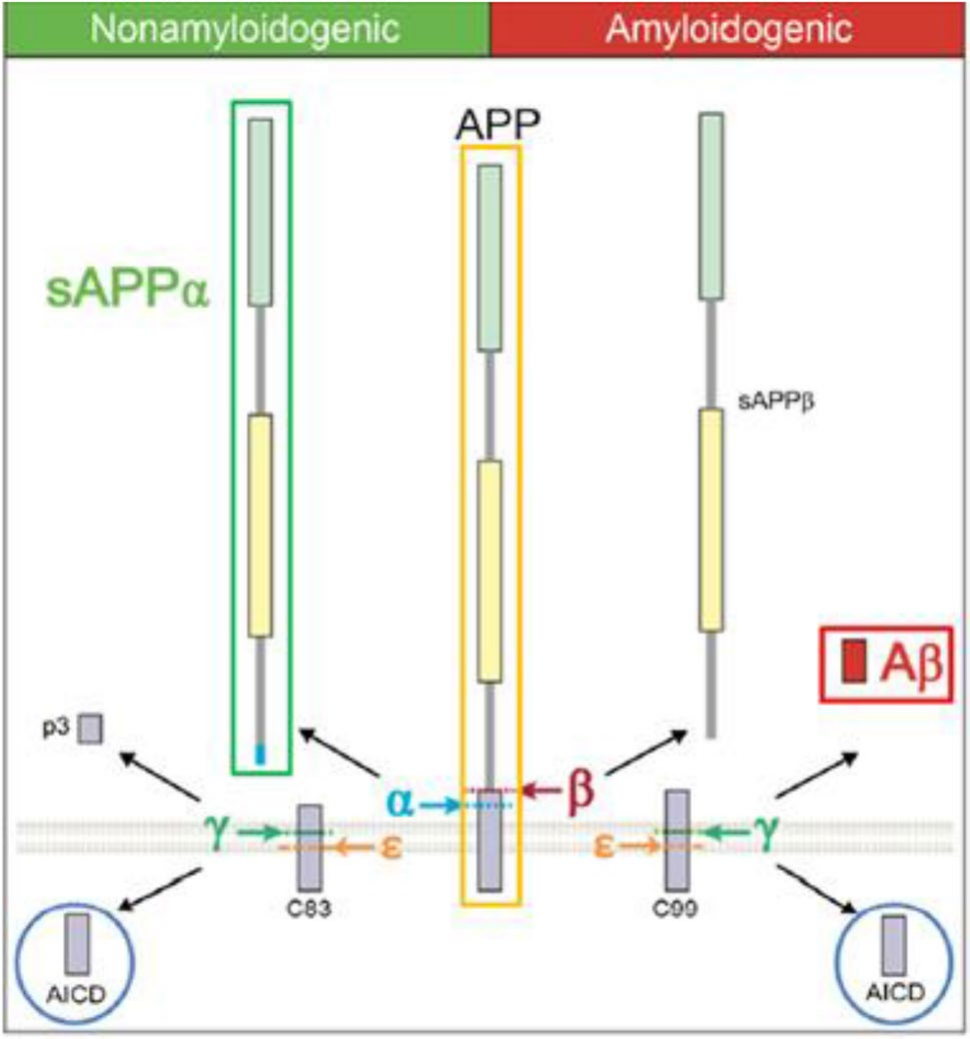
Cleavage of amyloid precursor protein (APP) by nonamyloidogenic and amyloidogenic pathways.

Pathologically, the primary characteristic of neuropathological lesions in AD is the extracellular deposition of amyloid plaques. Amyloid plaque aggregates are composed of amyloid-beta (Aβ), a fragment of amyloid precursor protein (APP) and a single transmembrane protein^1^. As in Fig. 1, APP is processed by two alternative pathways: nonamyloidogenic and amyloidogenic^2^. In the nonamyloidogenic pathway, APP is cleaved by α-secretase and γ-secretase generating the extracellular soluble APP-α (sAPP-α), APP intracellular domain (AICD) fragment and a short fragment p3 (N-truncated Aβ fragment)^3^. In the amyloidogenic pathway, at which Aβ fragments are produced, there are sequationuential cleavages by β- and γ-secretase. APP, at first, is cleaved by β-secretase-producing soluble APP-β (sAPP-β), and then the membrane-retained fragment is cleaved by γ-secretase generating. Another AICD fragment translocated to the nucleus where it affects the transcriptional regulation of several proteins and drives neuroprotective pathways and Aβ fragments of 40 (Aβ40) or 42 (Aβ42) amino acids interacting initially with apolipoprotein E result in an aggregation of beta oligomers to generate beta-amyloid plaques. Eventually, Aβ fragments are involved in several downstream pathways related to AD^4^.

Recently, researchers have introduced novel therapeutic approaches for AD that target the reduction of amyloid oligomer levels, including (1) the use of small molecule inhibitors to prevent oligomerization. (2) Employ the immunotherapy to neutralize oligomeric species. (3) Accurate determination of Aβ-degrading enzymes to dominate Aβ oligomer levels in the brain. (4) Stimulation of the immune system to produce Aβ antibodies to attack aggregates. (5) Use of Aβ blockers to block amyloid channels. All these approaches are currently under development in the preclinical research stages^5^. However, biological studies can reveal the initiation of the Aβ pathways before the outset of AD symptoms, which contributes to targeting studies of early stages of treatment and slowing disease progression. Early diagnosis of AD, therefore, is needed to provide adequate treatment and avoid deterioration stages^5^.

**Figure 2:**
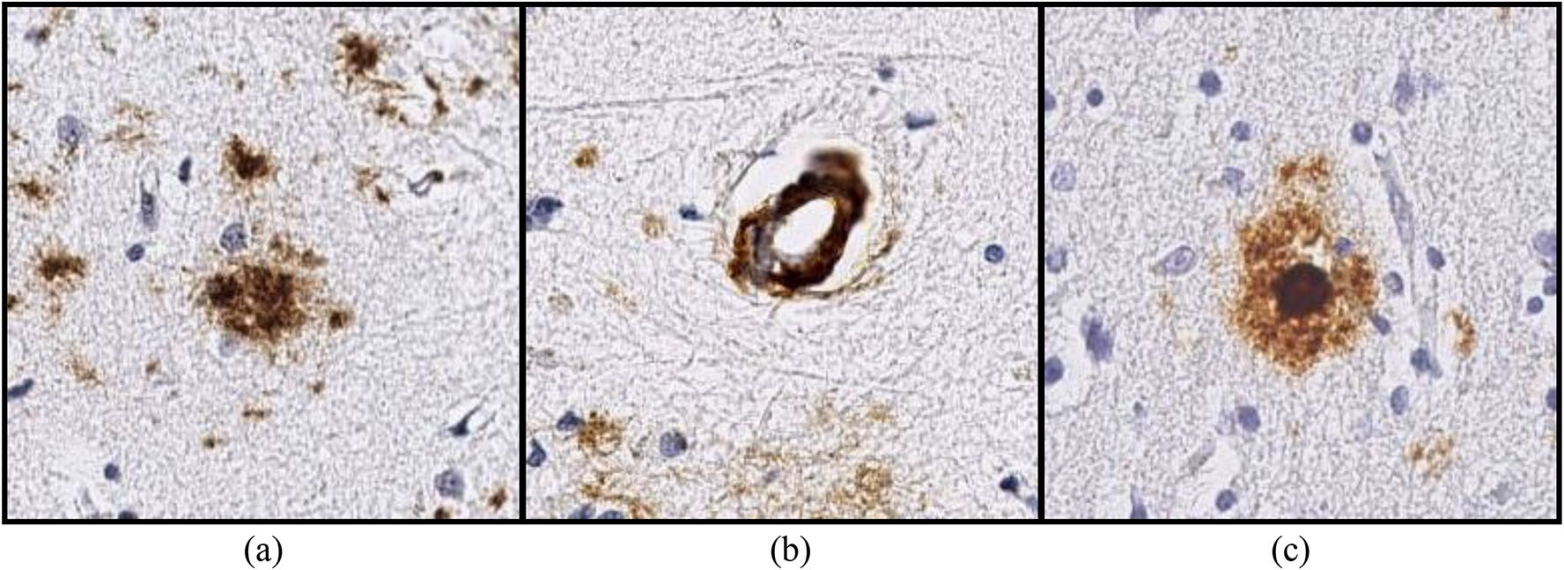
Amyloid-beta (Aβ) plaques morphologies: (**a**) diffuse, (**b**) cerebral amyloid angioplasty (CAA), and (**c**) dense-core.

Generally, the main challenge is not only to clear but also to prevent the formation of Aβ plaques requiring accurate measures of plaque morphologies for understanding disease progression and pathophysiology. Indeed, there are numerous forms of plaque, but the most prevalent form is characterized as a diffuse, cerebral amyloid angioplasty (CAA), and dense-core (see Fig. 2). The diffuse plaques are loosely organized amorphous clouds. Dense-core plaques are related to synaptic loss. They are surrounded by dystrophic neuritis, activated microglial cells, and reactive astrocytes. The dystrophic neurites are used for the pathological diagnosis of AD as they are associated with the presence of cognitive impairment. In CAA, the Aβ plaques deposit in the tunica media of leptomeningeal arteries and cortical capillaries, small arterioles, and medium-size arteries, particularly in posterior areas of the brain. Some degrees of CAA, usually mild ones, are presented in about 80% of AD patients. In case it is severe, CAA can weaken the vessel wall and cause life-threatening lobar hemorrhages^6^.

## Related works

The application of fractal geometry has become a new trend in studying biological systems in the last years^7–14^, including AD^15–20^. Fractal is an amorphous geometric concept with infinite nesting of a self-similar structure at different scales providing a general framework for studying different irregular sets. Fractal dimension (FD) seems to be a measure of the fractal properties that describe the space-filling properties of networks, including biological systems. FD has been applied to histopathological studies to determine the complexity of certain tissue components^21–24^. Biscetti et al.^25^ measured FD and other parameters of superficial capillary plexus (SCP), intermediate capillary plexus (ICP), deep capillary plexus (DCP), and choriocapillaris of subjects with mild cognitive impairment (MCI) due to AD and cognitively healthy controls (CN). They found that FD shows early vessel recruitment as a compensative mechanism at disease onset. The calculation of FD from optical coherence tomography angiography (OCT-A) is scanned to show the retinal vascular changes in subjects with AD, MCI, and CN shown in^26^. They found that FD decreases in elderly people and is lower in males.

The limitation of fractal analysis in describing more complex structures like Aβ plaques by one exponent FD can be solved by multifractal analysis. Multifractal is a generalization of fractal geometry when FD is not sufficient as it provides a spectrum of fractal dimensions FDs^27–29^. Multifractal measures have been observed in different physical situations as neural networks, fluid turbulence, rainfall distribution, mass distribution across the universe, viscous fingering, and many other phenomena.

Machine learning (ML) is a branch of artificial intelligence, which extracts information “training data” from a dataset to make accurate predictions or decisions without being explicitly programmed. Many studies have focused on applying machine learning techniques to diagnose and classify the various stages of AD via different types of physical tests in the last years^30–40^, and recently using immunohistochemistry images^41,42^. In^43^, they use the convolutional neural network (CNN) model on IHC images to classify between Aβ morphologies as dense core plaques, diffuse plaques, and CAA. The utilization of deep learning (DL) to differentiate tauopathies, including AD, progressive supranuclear palsy (PSP), corticobasal degeneration (CBD), and Pick's disease (PID), based on IHC images shown in^44^. Using MRI scans, Majumder et al.^45^ applied the artificial neural network (ANN) technique to distinguish between AD and cognitively normal (CN). Mild cognitive impairment (MCI) to Alzheimer's disease (AD) transition prediction was carried out, in^46^, using the ANN algorithm in MRI images. Additionally, Richhariya et al.^47^ classified between several stages as CN vs. AD, MCI versus AD, and CN vs. MCI using recursive feature elimination and SVM.

Therefore, the principal objective of the current research is to study the morphologies of amyloid plaques in AD using multifractal analysis that may represent a vital pathway for the increase in the number of neurodegenerative diseases, including Alzheimer's, as well as structure-based drug discovery, which may contribute to the creation of novel treatment strategies for various degenerative diseases. The variety of tissue structures in Whole-Slide Imaging (WSI) in the temporal gyri of the AD patient brain have been discussed in this research. To automate the classification process, the Naive Bayes has been used as a classifier.

### The research contributions

The current study contribution can be summed up as follows:

1. A new strategy in assessing of amyloid plaques morphologies using multifractal geometry of analysis.

2. Accurate measure of plaques morphologies for understanding disease pathology.

3. Using Naive Bayes classifier as a classifier saves time and effort other than algorithms that require training procedures.

4. It provides high performance measures compared with other recent classification techniques.

## Materials

Data used by Tang et al. are available at^48^. There are 63 subjects in the sample, and each has a single temporal gyri whole slide image (WSI). The subjects were chosen to represent a broad spectrum of pathological burden for each of the three AD pathologies of interest: cerebral amyloid angiopathy (CAA), dense-core and diffuse plaques. Glass slides with 5 mm sections of the superior and middle temporal gyrus that had been formalin-fixed and paraffin-embedded made up all of the WSIs. Amyloid beta (Aβ) antibody was used to perform immunohistochemistry staining on the tissue. An Aperio AT2 was used to digitize every slide at a magnification up to 40 times. The open-source library PyVips was used to apply the color normalization and subsequently tile the WSI into small images in a structured format (256 × 256 pixels). The used dataset contains 1200 images divided into 400 images for diffuse, 400 images for cerebral amyloid angioplasty (CAA), and 400 images for dense-core cases. Using a custom program written by MATLAB v.9.4 for R2018a (Mathworks, MA, USA), the hardware system is composed of a CPU core i7, 8GB RAM, and 1TB HD.

**Figure 3:**
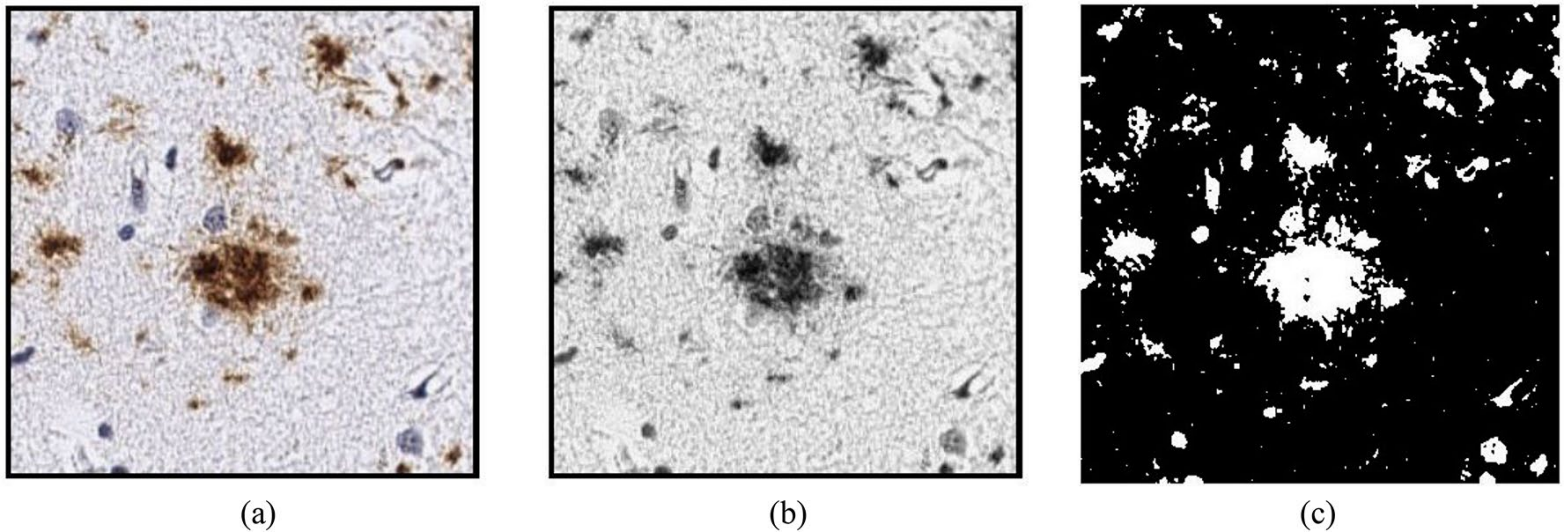
Sample fore image processing step (**a**) the raw image (**b**) the image in gray scale (**c**) the binary image.

In this study, the first step of the proposed classification system is the image-processing step. The images, firstly, have been processed to enhance the contrast and resolution. Secondly, the images have been passed through two processing stages: the first stage is responsible for converting the images from an RGB image to a Grayscale image. In the second stage, the images have been converted to a binary form; this can be illustrated in Figure 3. The binarization process is based on converting the image pixel level into two values 1 or 0; therefore, the resulting image has only two colors (Black and white). The pixel conversion process can be achieved through two steps. In the first step, obtain the image histogram, which describes the gray color distribution of the pixels in an image. In the second step, compute the threshold value according to the used threshold technique. In this study, Otsu’s method^49^ has been used as a threshold technique. This technique is based on maximizing the inter-cluster variation to minimize the intra-cluster variation; hence, it divides all the pixels into two clusters (foreground and background) based on the grayscale intensity values of the image pixels.

## Methods

### Multifractal analysis

In the last decades, a broad range of complex structures of interest to scientists, engineers, and physicians have been quantitatively characterized using the idea of a fractal dimension: a dimension that uniquely correlates to the geometrical shape under study and is often not an integer^50^. The key to this trend is the recognition that many random structures obey a symmetry as remarkable as that obeyed by regular structures. This "scale symmetry" implies that objects appear to be the same at many different scales of observation. To describe a fractal set, it is supposed that *S* is a subset of a d-dimensional space covered with boxes of length L, then the local density P_i_(L) of the object is the mass function of the i-th counting box,

1 $$ P_{i} \left( L \right) = \frac{{M_{i} \left( L \right)}}{{M_{T} }} $$

where M_T_ denotes the object's total mass and M_i_(L) is the number of pixels that comprise the mass in the box. On the other hand, P_i_(L) in heterogeneous objects can vary as:

2 $$ P_{i} \left( L \right) \sim L^{{\alpha_{i} }} $$

where α_i_ is the Holder exponent that characterizes the scaling of the i-th region or spatial location. Consequently, the local behavior of P_i_(L) around the center of a counting box with length *L* is thus demonstrated by α_i_. The number of boxes N(α) where the mass function has exponents range between α and α + dα scales as:

3 $$ M\left( \alpha \right) \sim L^{ - f \left( \alpha \right)} $$

where f(α) is the fractal dimension of the fractal units at particular sizes. Scaling of the q-th moments of the density function P_i_(L) yields to multifractal measures as

4 $$ \sum\nolimits_{i = 1}^{M(L)} {P_{i}^{q} (L) = L^{{(q - 1)D_{q} }} } $$

Hence, the exponent in Eq. (4) is called the mass exponent of q-th moment of order τ(q) that admits the following equation:

5 $$ \tau \left( q \right) = \left( {q - 1} \right)D_{q} $$

It is well known as:

6 $$ \alpha \left( q \right) = \frac{d\tau \left( q \right)}{{dq}} $$

where D_q_ denotes the generalized dimensions defined as:

7 $$ D_{q} = \frac{1}{q - 1}\mathop {\lim }\limits_{L \to 0} \frac{{\ln \;\sum\nolimits_{i = 1}^{M(L)} {P_{i} (L)^{q} } }}{\ln (L)} $$

**Figure 4:**
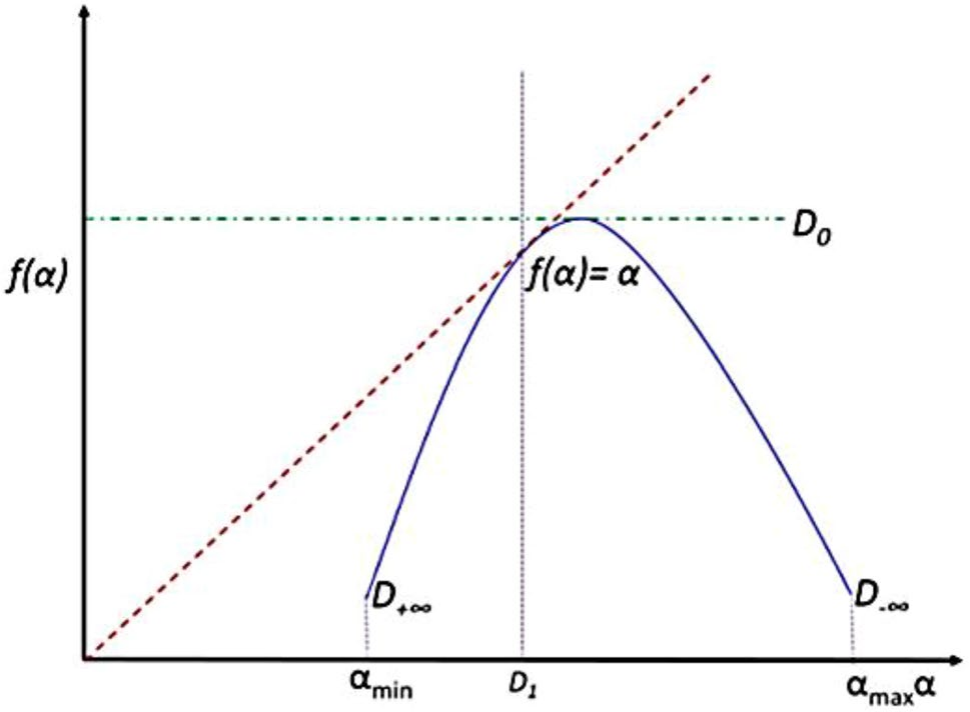
The singularity spectrum.

The multifractal spectrum illustrated in Fig. 4 is a convex function with a maximum D_o_ at q = 0 and is known as the box-counting dimension^51^. For q = 1, f (α) = α = D_1_ is the information dimension. D_1_ represents the scaling of information generation that describes the rate of information gain by successive measurements or the rate of information loss by time^52^.

In fact, the set of local scales that may be stated as powers of *L* is the only one used to estimate the multifractal spectrum because it cannot be calculated as infinity. Additionally, this fact limits the variety of moment q that can be applied^53^. Therefore, the multifractal spectrum can be computed from:

8 $$ \mu_{i} \left( {q, \;L} \right) = \frac{{ P_{i}^{q} \left( L \right)}}{{\mathop \sum \nolimits_{i = 1}^{M\left( L \right)} P_{i}^{q} \left( L \right)}} $$

Thus, the computation of f (q) and α(q) goes as follows:

9 $$ f\left( q \right) = \mathop {\lim }\limits_{L \to 0} \frac{H\left( L \right)}{{\log L}} = \mathop {\lim }\limits_{L \to 0} \frac{{\mathop \sum \nolimits_{i = 1}^{M\left( L \right)} \mu_{i} \left( {q,\;L} \right)^{ } \log \mu_{i} \left( {q,\;L} \right)}}{\log L} $$

And

10 $$ \alpha \left( q \right) = \mathop {\lim }\limits_{L \to 0} \frac{W\left( L \right)}{{\log L}} = \mathop {\lim }\limits_{L \to 0} \frac{{\mathop \sum \nolimits_{i = 1}^{M\left( L \right)} \mu_{i} \left( {q,\;L} \right)^{ } \log P_{i} \left( L \right)}}{\log L} $$

**Figure 5:**
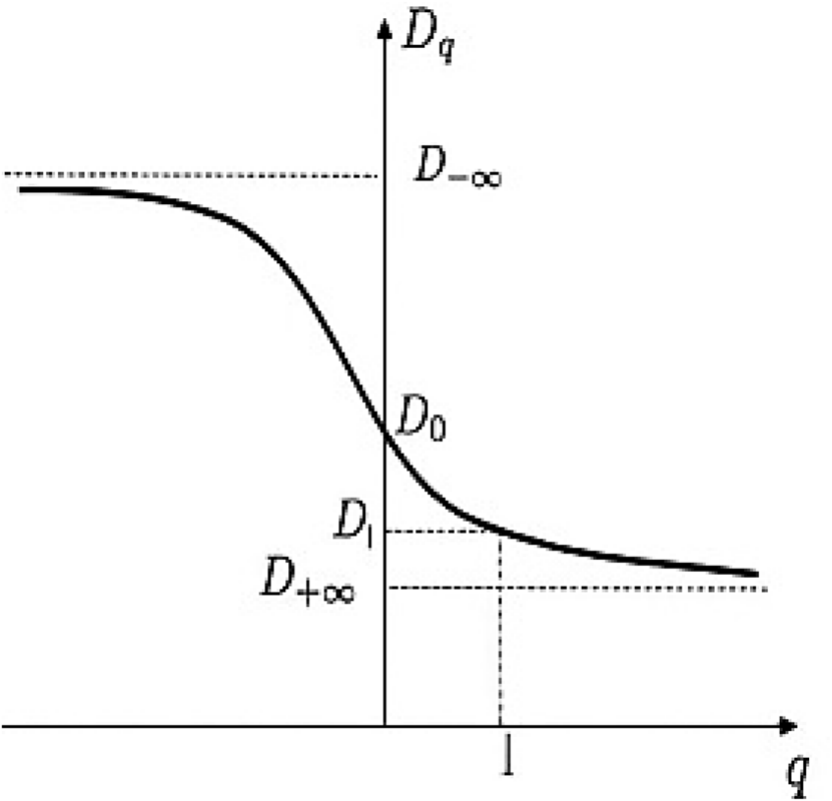
The multifractal generalized dimension.

The second commonly used graph discussed here is the generalized dimension curve (D_q_ vs. q), which is analogous to applying warping filters to an image to exaggerate parameters that might otherwise be unnoticeable. The term "warp filters" refers to a group of arbitrary exponents represented by the symbol "q". Hence, we can construct a generalized dimension Dq for each q as shown in Fig. 5.

The generalized dimension D_q_ can be defined as:

11 $$ D_{q} = \frac{1}{1 - q}\mathop {\lim }\limits_{L \to 0} \frac{{\ln I\left( {q,\;L} \right)}}{{{\text{ln}}\left( {1/L} \right)}} $$

where I (q, r) is the partition function given by:

12 $$ I\left( {q,\;L} \right) = \ln \mathop \sum \limits_{i = 1}^{N\left( L \right)} P_{i} \left( L \right)^{q} $$

Equation (11) becomes:

13 $$ D_{q} = \frac{1}{1 - q}\mathop {\lim }\limits_{L \to 0} \frac{{\ln \mathop \sum \nolimits_{i = 1}^{N\left( L \right)} P_{i} \left( L \right)^{q} }}{{\ln \left( {1/L} \right)}} $$

where r denotes the scale of measurement, q is the order of the moment, N(L) is the number of fractal copies based on the scale L and P_i_(L) is the growth probability function of the *i*-th fractal unit. From the general dimension definition, at q = 0, D_o_ describe the box-counting dimension (D_B_), also known as the capacity dimension. In Eq. (13), when we use a grid of boxes to cover a given space, the box-counting dimension D_0_ is given by:

14 $$ D_{0} = \mathop {\lim }\limits_{L \to 0} \frac{\ln N\left( L \right)}{{\ln \left( {1/L} \right)}} $$

When N(L) is the number of nonempty boxes with length L that cover the space and include at least some part of the attractor (not necessarily the total number of points). At q = 1, D_1_ is the information dimension (D_I_) that characterizes the rate of information loss by time or the rate of information gain by sequential measurements. D_I_ analogous to a quantity known as the Shannon entropy. It is given by:

15 $$ H\left( L \right) = - \mathop \sum \limits_{i = 1}^{N\left( L \right)} P_{i } \left( {\text{L}} \right){\text{ ln}}P_{i} \left( L \right) $$

Provided we apply the Taylor expansion to Eq. (12), we have:

16 $$ \ln I\left( {q,L} \right) = \left( {q - 1} \right)ln\mathop \sum \limits_{i = 1}^{N\left( L \right)} P_{i} \left( L \right)\ln P_{i} \left( L \right) $$

So, Eq. (13) becomes:

17 $$ D_{I} = \mathop {\lim }\limits_{L \to 0} \frac{{\ln \mathop \sum \nolimits_{i = 1}^{N\left( L \right)} P_{i} \left( L \right)\ln P_{i} \left( L \right)}}{{\ln \left( {1/L} \right)}} $$

At q = 2, D_2_ is the correlation dimension^54^, which characterizes the correlation between pairs of points on a reconstructed attractor. From Eq. (13), the correlation dimension (D_C_) is given by:

18 $$ D_{C} = \mathop {\lim }\limits_{L \to 0} \frac{{\ln \mathop \sum \nolimits_{i = 1}^{N\left( L \right)} P_{i} \left( L \right)^{2} }}{lnL} $$

If D_0_ = D_1_ = D_2_, the structure is termed as monofractal or unifractal. If D_o_ > D_1_ > D_2_, the structure is termed as multifractals.

### Lacunarity measurement

Lacunarity is a measure of the different gaps distribution throughout an image^55^. It gives an assessment of the structure heterogeneity. The higher lacunarity value, the less heterogeneous in the fractal geometry. The mean lacunarity Λ can be written as:

19 $$ {\Lambda } = \left( {\sigma /\mu } \right)^{2} $$

where *µ*: the mean for pixels per box*, σ*: the standard deviation.

### Naïve Bayes

It is a supervised learning algorithm based on Bayes’ theorem. Naïve Bayes is considered as a probabilistic classifier with an assumption of independence among predictors. It has several advantages: (1) Fast, easy and simple to implement. (2) No need for large training datasets. (3) It can be used for discrete and analogue data. The main idea in the Naive Bayes classifier is that the presence of a particular feature is unrelated to the presence of any other features. Therefore, it cannot be learnt if there is a relation between the features^56–58^.

Bayes' theorem is used to determine the probability of a hypothesis with the prior knowledge of a class. It can be described by:

20 $$ P\left( {C{|}x} \right) = \frac{{P\left( {x{|}c} \right)P\left( C \right)}}{P\left( x \right)} $$

where $P\left(C|x\right)$ "Posterior probability": is the probability of hypothesis/class *"C"* on the observed event/features *"x"*; $P(C)$ "Prior probability": is the probability of hypothesis before observing the evidence. $P\left(x|C\right)$ "Likelihood probability": is the probability of the evidence given that the probability of a hypothesis is true. $P(x)$ "Marginal Probability": is the probability of the evidence or the prior probability of predictor.

Assuming that ***X*** represents as the extracted features and can be written as:

21 $$ X = \left( {x_{1} ,\;x_{2} ,\;x_{3} ,\; \ldots .,\;x_{n} } \right) $$

Therefore, the probability of a hypothesis/class ***c*** for the selected features ***X*** with number ***n*** can be written as:

22 $$ P\left( {C{|}x_{1} ,\;x_{2} ,\;x_{3} ,\; \ldots ,\;x_{n} } \right) = \frac{{P\left( {x_{1} {|}c} \right)P\left( {x_{2} {|}c} \right)P\left( {x_{3} {|}c} \right) \ldots P\left( {x_{n} {|}c} \right)P\left( C \right)}}{{P\left( {x_{1} } \right)P\left( {x_{2} } \right)P\left( {x_{3} } \right) \ldots P\left( {x_{n} } \right)}} $$

Equation (22) can be written in simple form as:

23 $$ P\left( {C{|}x_{1} ,\;x_{2} ,\;x_{3} ,\; \ldots ,\;x_{n} } \right) = \frac{{P\left( C \right)\mathop \prod \nolimits_{i = 1}^{n} P\left( {x_{i} {|}c} \right)}}{{\mathop \prod \nolimits_{i = 1}^{n} P\left( {x_{i} } \right)}} $$

According to the used datasets, the classifier system may have ***m*** classes:

24 $$ C = \left( {c_{1} ,\;c_{2} ,\;c_{3} ,\; \ldots ,\;c_{m} } \right) $$

Then the classifier system can select the class with the highest probability value as:

25 $$ C = argmax_{j = 1}^{m} = \frac{{P\left( {c_{j} } \right)\mathop \prod \nolimits_{i = 1}^{n} P\left( {x_{i} {|}c_{j} } \right)}}{{\mathop \prod \nolimits_{i = 1}^{n} P\left( {x_{i} } \right)}} $$

In this study, there are three classes (*m* = 3) of Aβ plaques $\left({c}_{1},{c}_{2},{c}_{3}\right)$ as diffuse, CAA, and dense-core. The RF optimized hyperparameters^59^ can be listed in Table 1.

**Table 1:** The RF optimized hyperparameters.

Number of decision trees	120
Sampling data points method	Bootstrap
The quality measure of a split	“Gini function”
The features number for the best split	max_feature
The needed number of samples to be at a leaf node	min_samples_leaf
The tree maximum levels	max_leaf_nodes = 22

**Figure 6:**
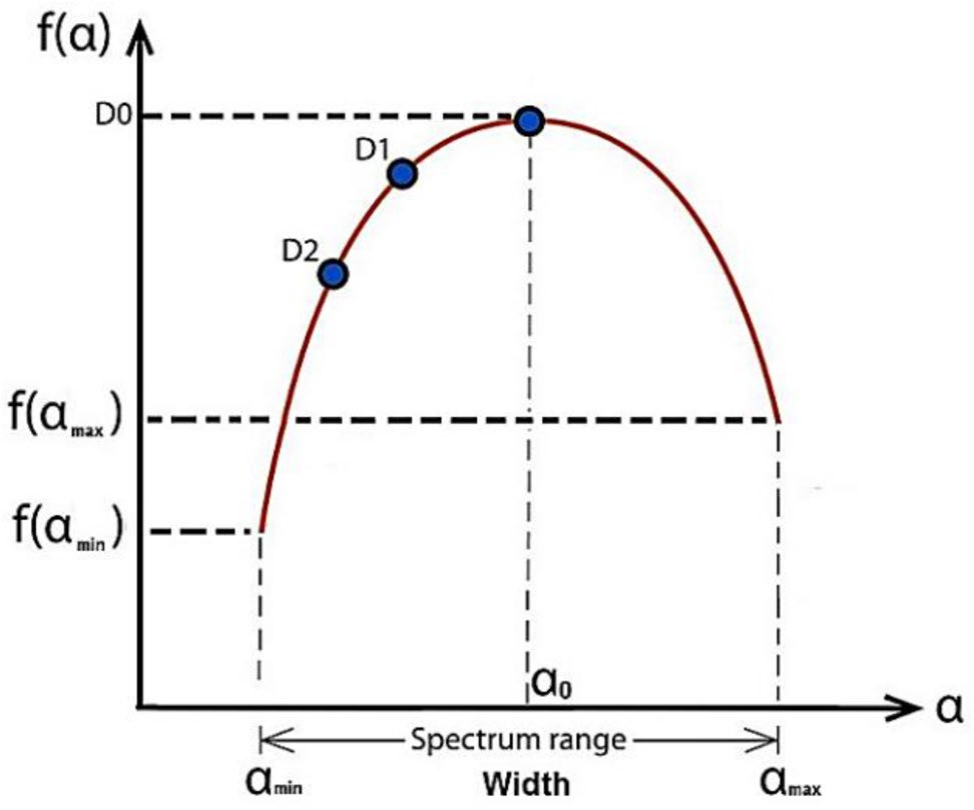
The extracted features.

The methodology is based on extracting the most changeable features related to AD, the system has 12 extracted features (*X* = 12). These features can be illustrated in Fig. 6 and listed as follows:

1. The lacunarity (λ),
2. The maximum value of α (α_max_) in the singularity spectrum,
3. The singularity spectrum at the α_max_ (f(α_max_)),
4. The minimum value of α (α_min_) in the singularity spectrum,
5. The singularity spectrum at the α_min_ (f(α_min_)),
6. The α value at the maximum of the singularity spectrum curve (α_0_),
7. The width of the singularity spectrum curve (width),
8. The symmetrical shift of the singularity spectrum curve,
9. The box-counting dimension (D_0_),
10. The Information dimension (D_1_),
11. The correlation dimension (D_2_).

These features can be illustrated in Fig. 6

Most of the time, reducing the number of input variables or the extracted features might enhance the efficiency of the model, as well as lowering the computing cost of modelling. Therefore, when creating a predictive model, it is desired to perform a feature selection process to reduce the number of extracted features. This can be done by using a feature selection algorithm as a Random Forest (RF) algorithm^60,61^.

### Random forest algorithm

**Figure 7:**
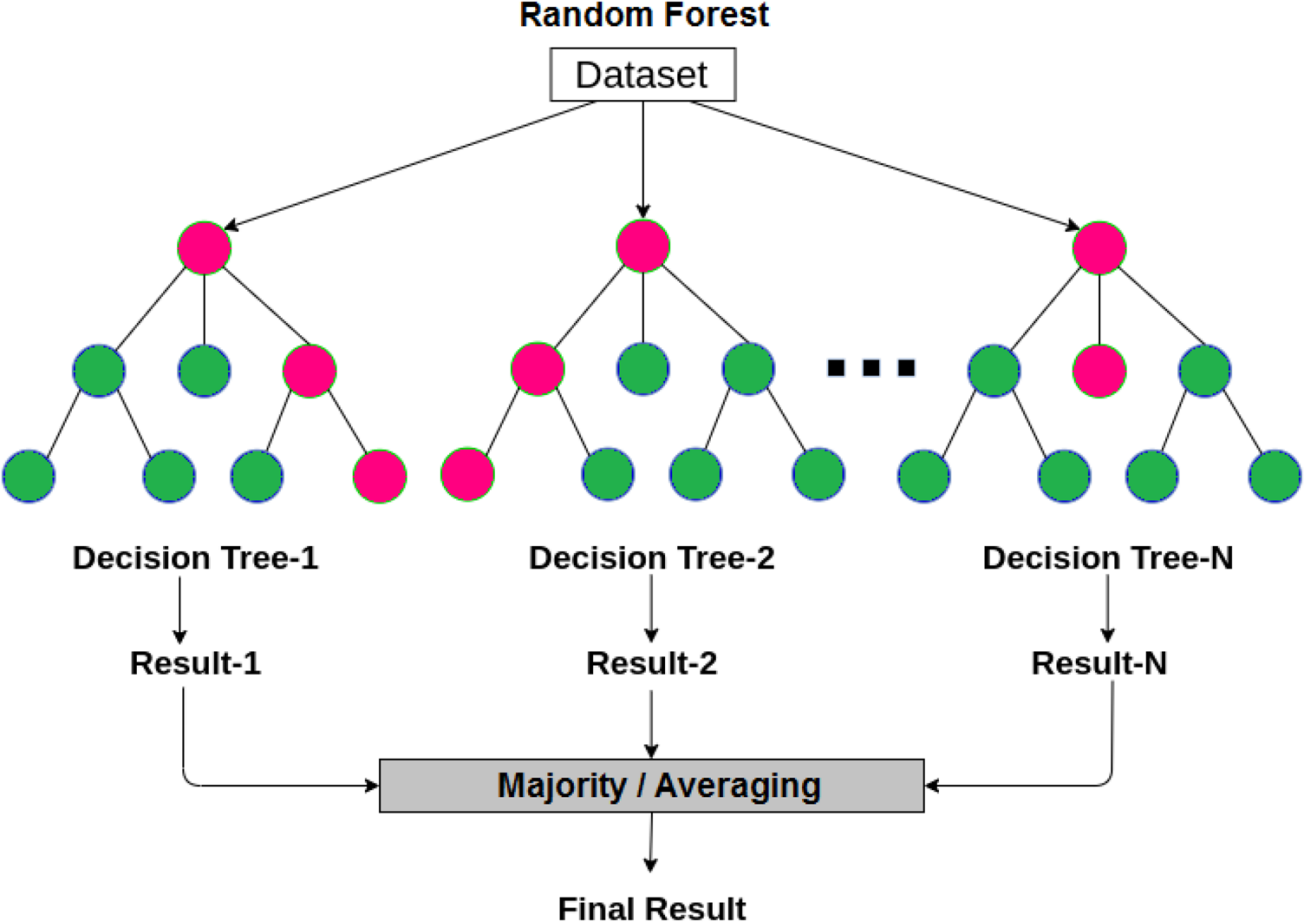
The random forest algorithm.

Random forest is a supervised machine learning algorithm. It is a modified version of the decision trees. It is usually trained using the “bagging” method. It is a collection of multiple decision trees to increase the overall result. To start the training of the RF algorithm, three parameters have to be adjusted first to be operated as a classifier procedure. These parameters can be summarized as (1) the number of the used trees, (2) the number of nodes, and (3) the number of the features sampled. As shown in Fig. 7.

Several advantages can be obtained as a result of using the RF algorithm, these advantages can be listed as (1) reducing the risk of overfitting, (2) performing both classification and regression tasks, (3) giving a good explanation for the resultant, (4) easily determination of the important features, and (5) easily handling of large datasets. However, RF suffers from disadvantages as (1) large time-consuming, (2) more computation resources, and (3) more complex in prediction than the decision tree.

In almost all classification systems, hundreds or thousands of features are used to obtain accurate results. On the other hand, not all the extracted features are important or play a strong influence in the classification processes. Therefore, it is required to create a classification model that includes the most important features, called "Feature Selection". This makes the model simpler, reduces the computational time, and reduces the model variance.

The feature selection can be performed by using a Recursive Feature Elimination procedure^62,63^. In this study, after creating the classification model, the less relevant feature is removed. Features are ranked by the model performance measures, eliminating the less important features per loop. Repeat the procedures until reaches the high-ranked features.

**Figure 8:**
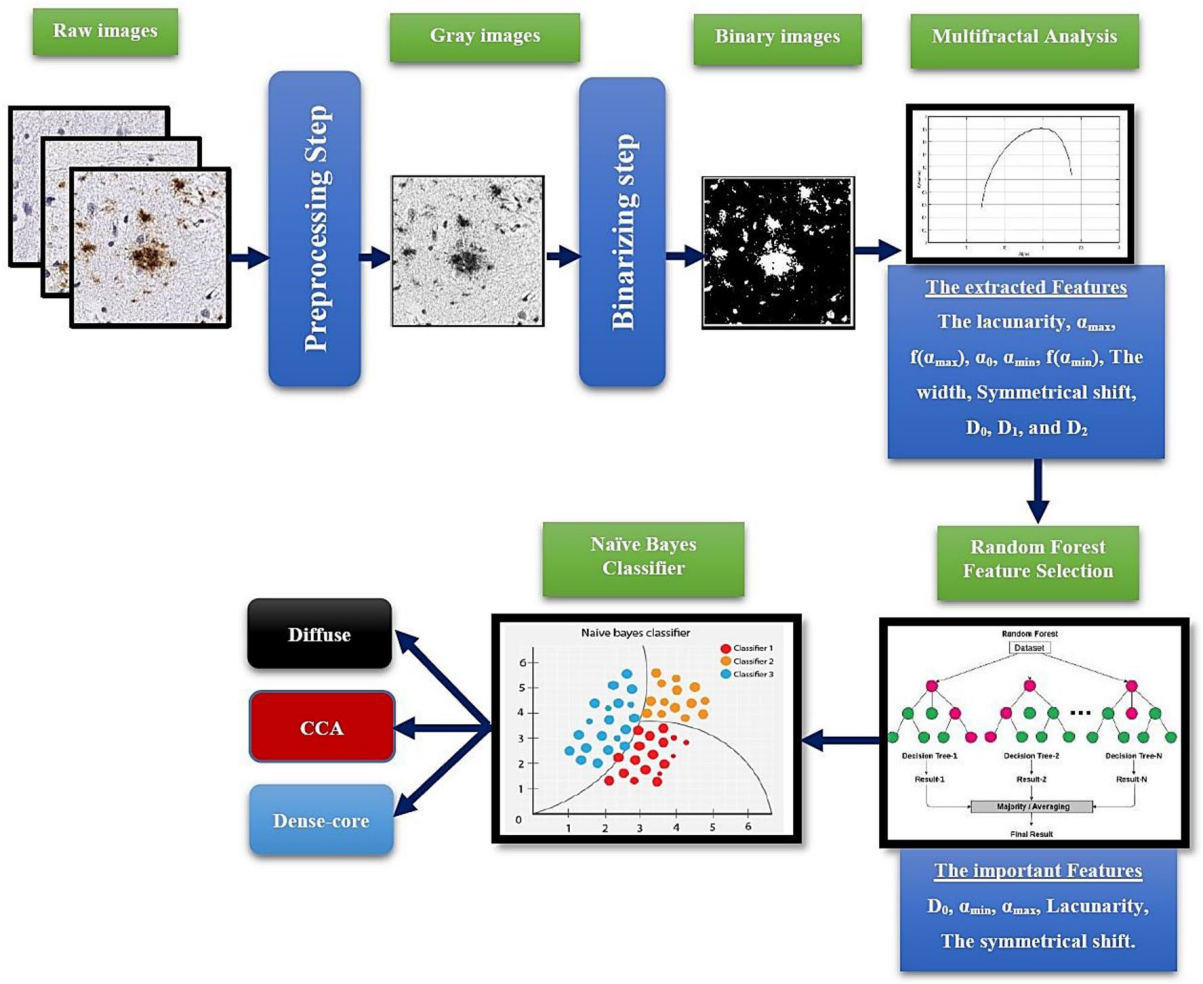
The workflow of the proposed methodology.

The workflow of the proposed methodology can be summarized as shown in Fig. 8.

## Results and discussions

### The dataset demographic characteristics

The proposed methodology based on using the archived images in Davis Alzheimer’s Disease Center Brain Bank^64^ at California University. These samples had the following features:

1. In order to ride of endogenous protein, the samples were pretreated with formic acid.
2. An amyloid-β antibody had been used to stain the tissue.
3. The samples were 5 μm formalin fixed
4. Portions of the human brain's superior and middle temporal gyrus that had been encased in paraffin.
5. Aperio Digital Pathology Slide Scanners were used for digitalizing the slides with magnification factor up to 40x.

The dataset demographic characteristics can be summarized in Table 2.

**Table 2:** The demographic characteristics.

Type	Samples	Training samples (75%)	Testing samples (25%)
Diffuse	400	300	100
CCA	400	300	100
dense-core plaques	400	300	100
Total of samples	1200	900	300

### The image singularity spectrums

**Figure 9:**
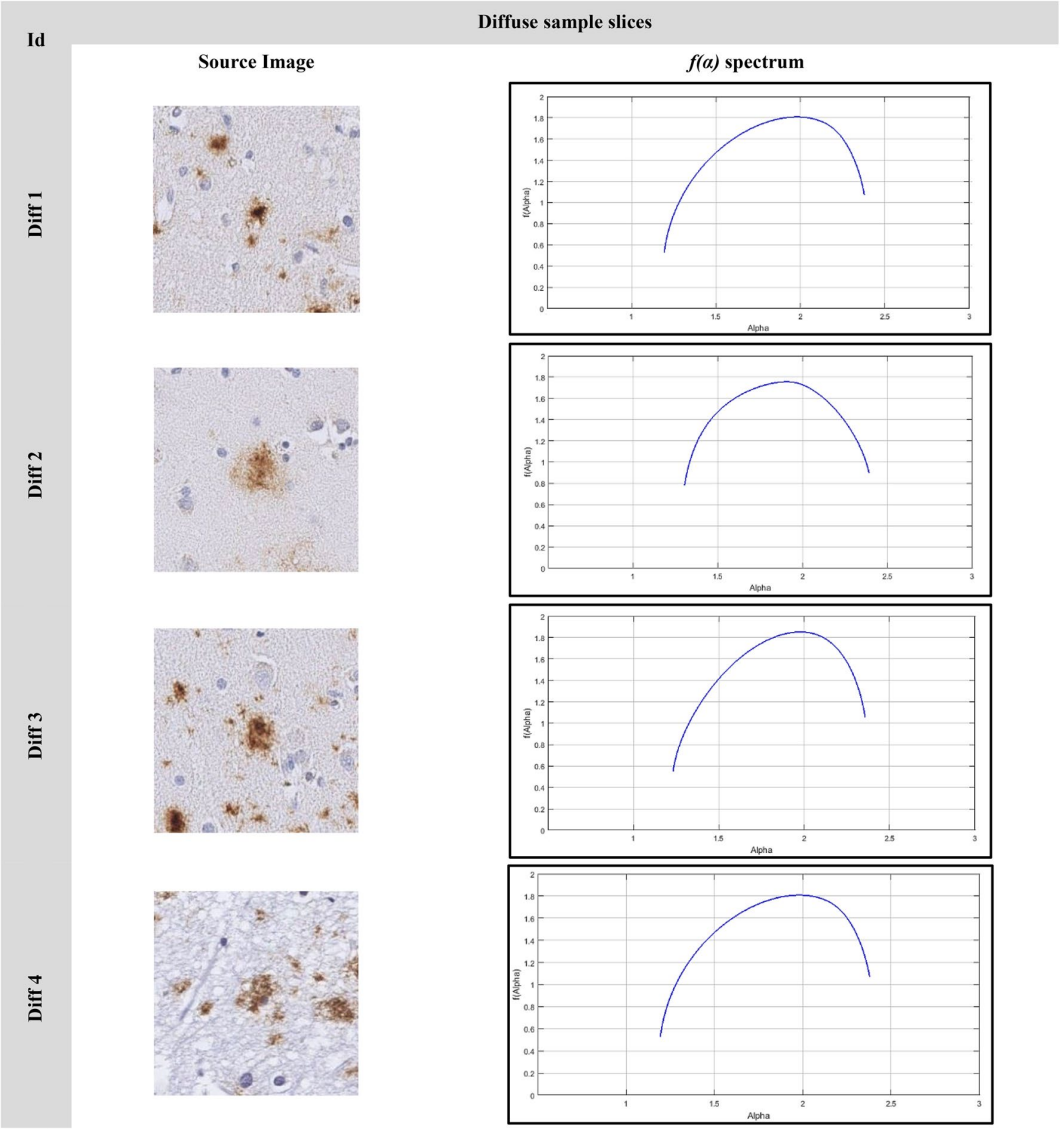
The Diffuse images singularity spectrum.

**Figure 10:**
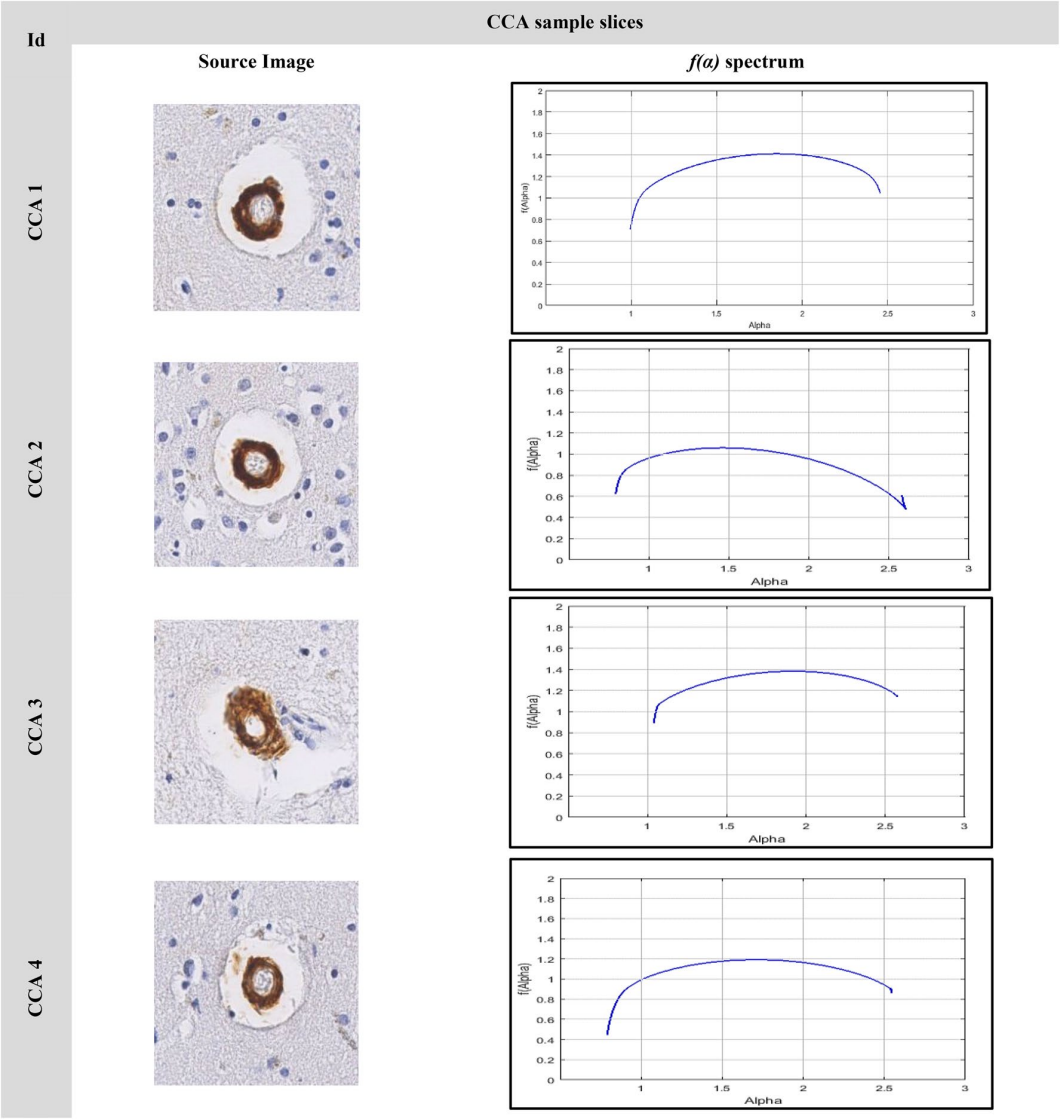
The CCA images singularity spectrum.

**Figure 11:**
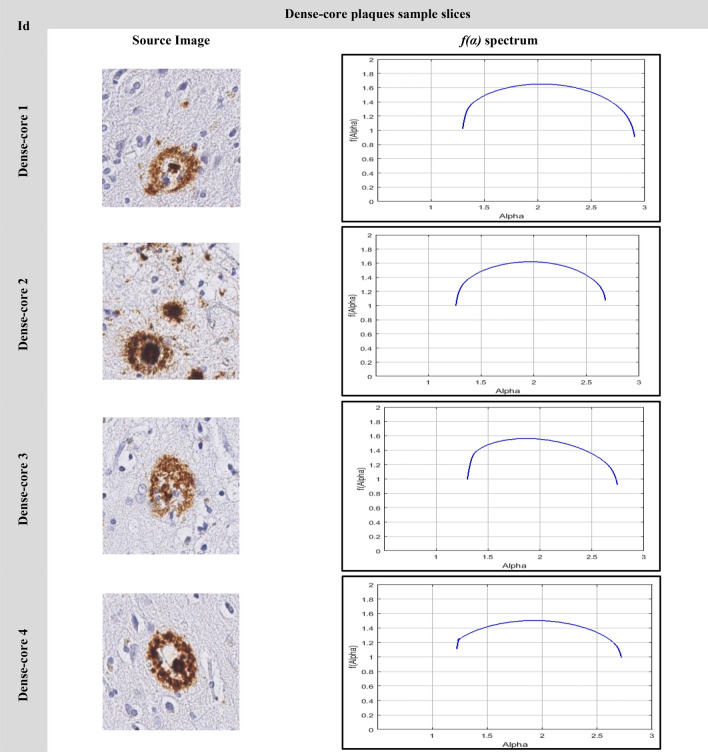
The Dense-core images singularity spectrum.

**Figure 12:**
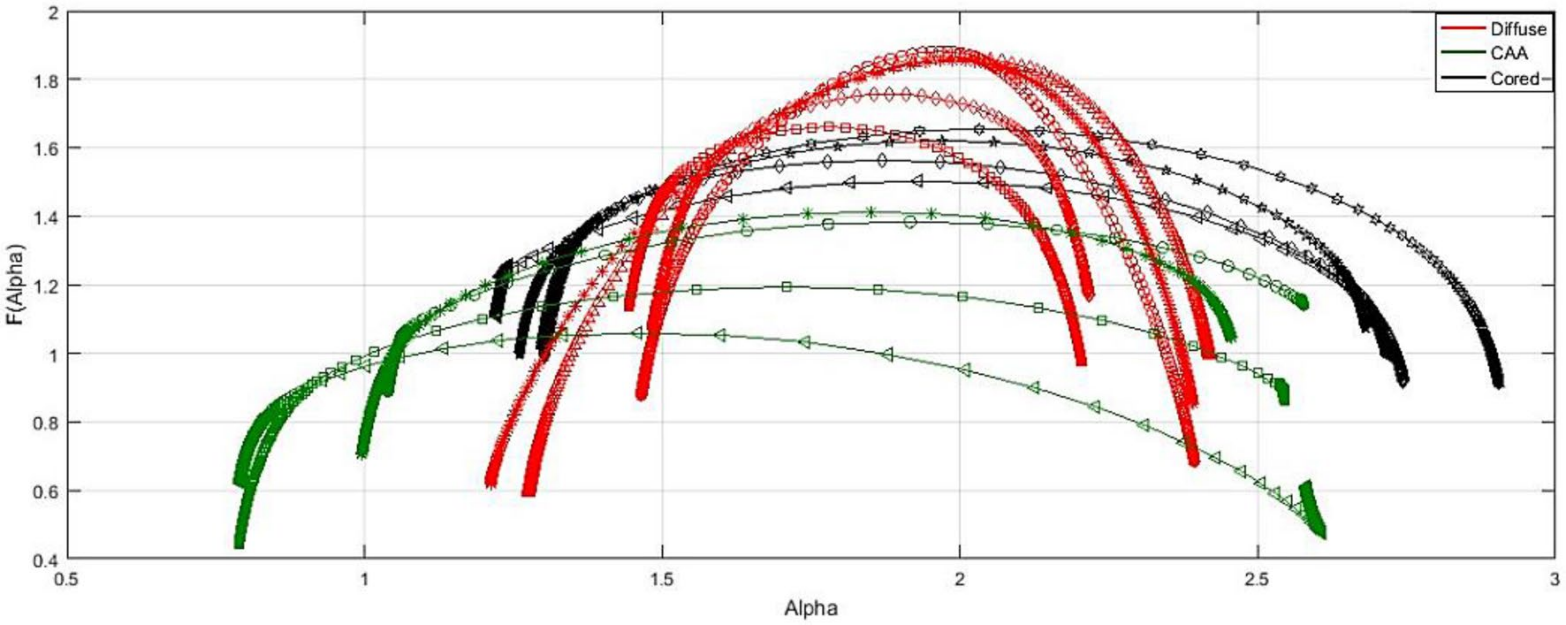
The singularity spectra for the AD stages.

The image analyses using multifractal are shown in Figs. 9, 10, 11. Figure 9 shows the singularity spectrum for diffuse cases. Figure 10 shows the singularity spectrum for the CAA cases. Figure 11 shows the singularity spectrum for the Dense-core cases. As the amyloid plaques increase, the heterogeneity in the brain tissue increases. Therefore, the spectrum became wider with different asymmetrical shapes as shown in Fig. 12. As the amyloid plaques increase, the curves have moved to the right as the image heterogeneities have grown, with differing singularity spectrum start and end values α_min_ and α_max_ respectively. Table 3 summarizes 15 sample images for AD with the extracted feature values.

**Table 3:** Sample of the extracted features data.

Image	D(Q)			f(α)							Lacunarity
Id	D_0_	D_1_	D_2_	α_max_	f(α_max_)	α_0_	α_min_	f(α_min_)	Width	Symmetric shift	
Diff 1	1.4499	1.2395	1.1631	2.3671	1.0132	1.7631	1.0142	0.7126	1.3529	−0.07245	1.0913
Diff 2	1.6481	1.1189	1.0778	2.0758	0.5581	1.4377	0.9654	0.5881	1.1104	0.0829	1.2665
Diff 3	1.5035	1.3747	1.2796	2.359	0.3601	1.6402	1.0726	0.6333	1.2864	0.0756	0.6524
Diff 4	1.5738	1.1757	0.9652	2.1782	1.4797	1.9434	0.7155	0.259	1.4627	−0.09655	2.885
Diff 5	1.7157	1.5076	1.3894	2.271	1.4144	1.9425	1.163	0.6775	1.108	−0.0255	1.4289
CCA 1	1.4126	1.145	1.0916	2.4546	1.0451	1.85	0.9955	0.7055	1.4591	−0.12495	1.0051
CCA 2	1.0602	0.878	0.8517	2.5816	0.6126	1.6599	0.7921	0.6203	1.7895	0.22695	0.5611
CCA 3	1.3836	1.098	1.0701	2.5736	1.1561	1.9161	1.0392	0.8893	1.5344	−0.2097	1.0921
CCA 4	1.3168	1.1295	1.0189	2.3581	1.2853	1.9803	0.9182	0.6754	1.4399	−0.34215	2.8685
CCA 5	1.1931	0.9439	0.9035	2.5453	0.8607	1.7091	0.7891	0.4433	1.7562	−0.1419	0.7133
Dense-core 1	1.3951	1.2956	1.293	2.7623	0.53	1.6265	1.2698	1.0551	1.4925	0.38955	0.5713
Dense-core 2	1.5864	1.5693	1.5389	2.6562	0.9811	1.9011	1.4546	1.1626	1.2016	0.1543	0.9127
Dense-core 3	1.4524	1.4306	1.3833	2.9044	0.9105	1.0321	1.2964	1.0203	1.608	0.1683	0.7929
Dense-core 4	1.563	1.4087	1.3803	2.7442	0.9178	1.8684	1.3012	0.9911	1.443	0.1543	0.6149
Dense-core 5	1.4206	1.4081	1.3552	2.6806	1.074	1.9691	1.2599	0.9974	1.4207	0.115	0.8102

**Figure 13:**
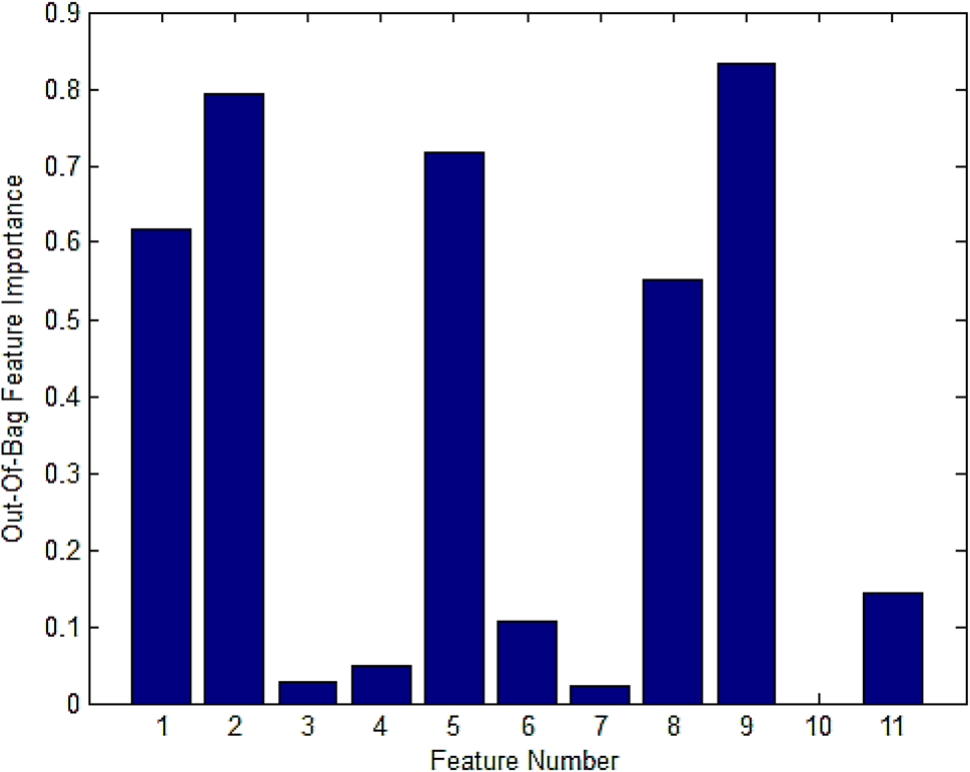
The features importance using RF algorithm 1) The lacunarity, 2) α_max_, 3)f(α_max_), 4) α_0_, 5) α_min_, 6) f(α_min_), 7) The width, 8) Symmetrical shift, 9)D_0_, 10) D_1_, and 11) D_2_.

**Figure 14:**
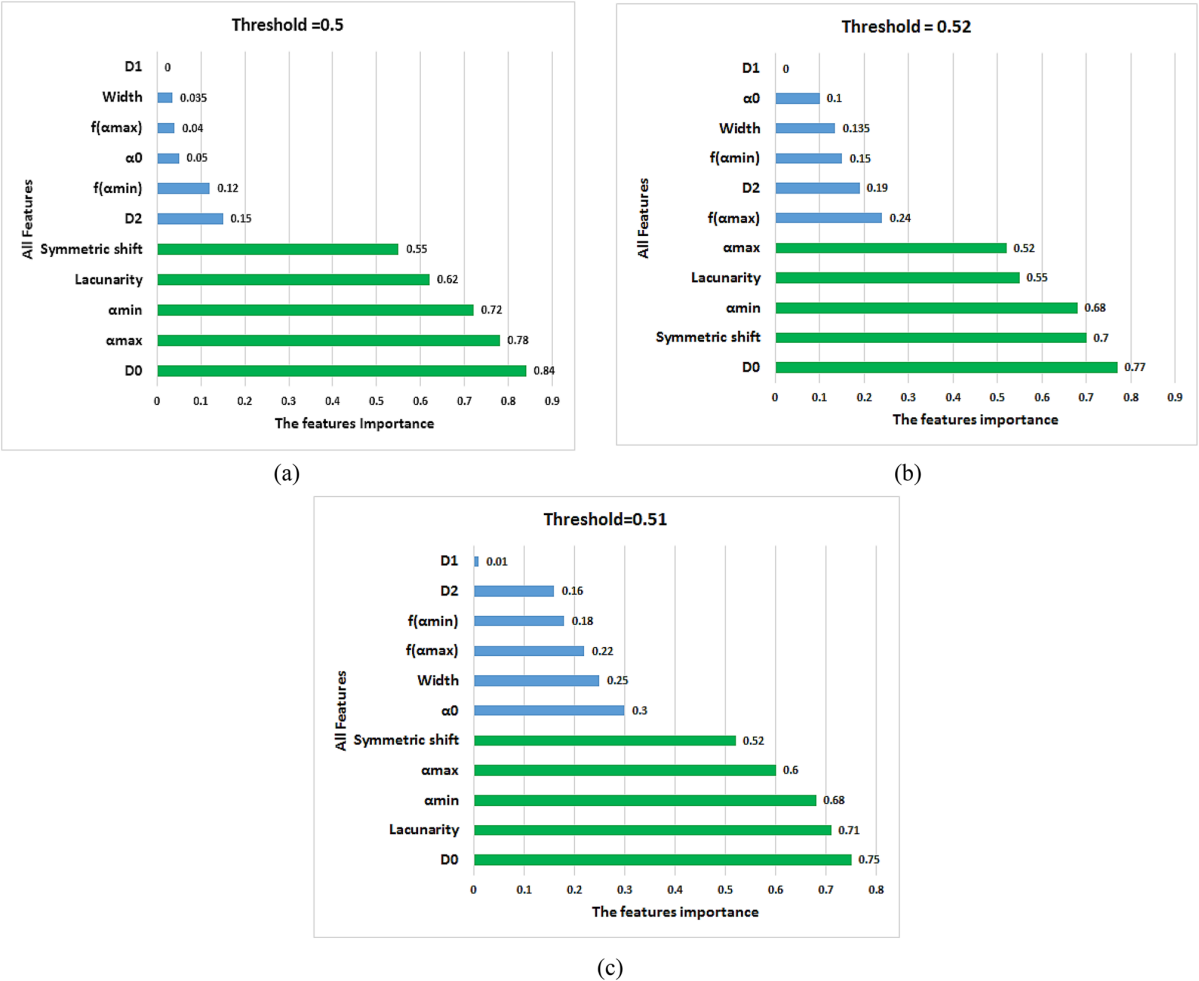
Raking of the feature importance provided by RF.

**Figure 15:**
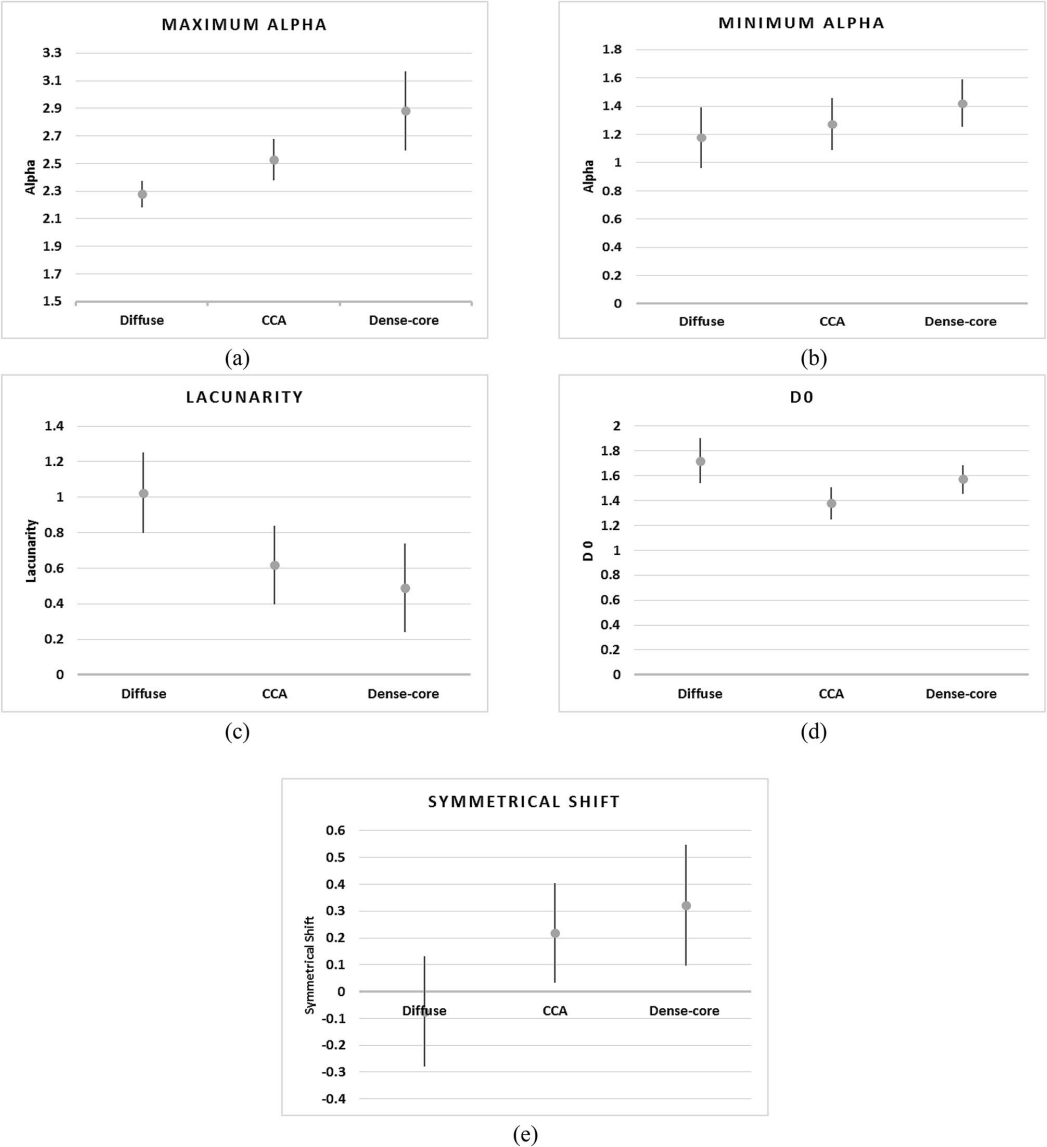
The statistical representation of the most important features of the AD stages.

According to the proposed methodology, eleven features have been extracted; they described the changes in the brain tissue related to AD. To reduce the used features, the RF algorithm is employed to remove the less relevant features and is described in Fig. 13. According to Fig. 13; the important features can be concluded as lacunarity, α_max_, α_min_, Symmetrical shift, and D_0._ They have an importance weight of not less than 0.5.

The Figure 14 shows the ranking of the feature importance provided by RF^59^. It represents the raking of the feature importance for the diffuse cases, CCA, and dense-core cases for different thresholds. The blue pars (features) are discarded as being under the threshold value. Performing a model evaluation using multiple thresholds, the optimum threshold value can be chosen as 0.5, due to the lack of importance of the discarded features as f(α_max_), α_0_, f(α_min_), The width, D_1_, and D_2_.

To explain the importance of the selected features, Figure 15 illustrates the statistical representation of the most important features.

As shown in Figure 15a,b, the diffuse stage has the lowest values of (αmax) and (αmin) while the dense-core stage has the largest value due to the increase in the amyloid plaques accumulation. In Figure 15c,d, the diffuse stage has achieved the highest (D_0_) and (Lacunarity) due to fewer Amyloid-beta plaques, which resulted from more homogeneity in the diffuse dataset images than other stages. As illustrated in Figure 13 and 15e, the diffuse stage has a shift left to the symmetrical axis of the singularity spectrum rather than CCA and dense-core stages have a shift right to the symmetrical axis.

## Performance measures

**Figure 16:**
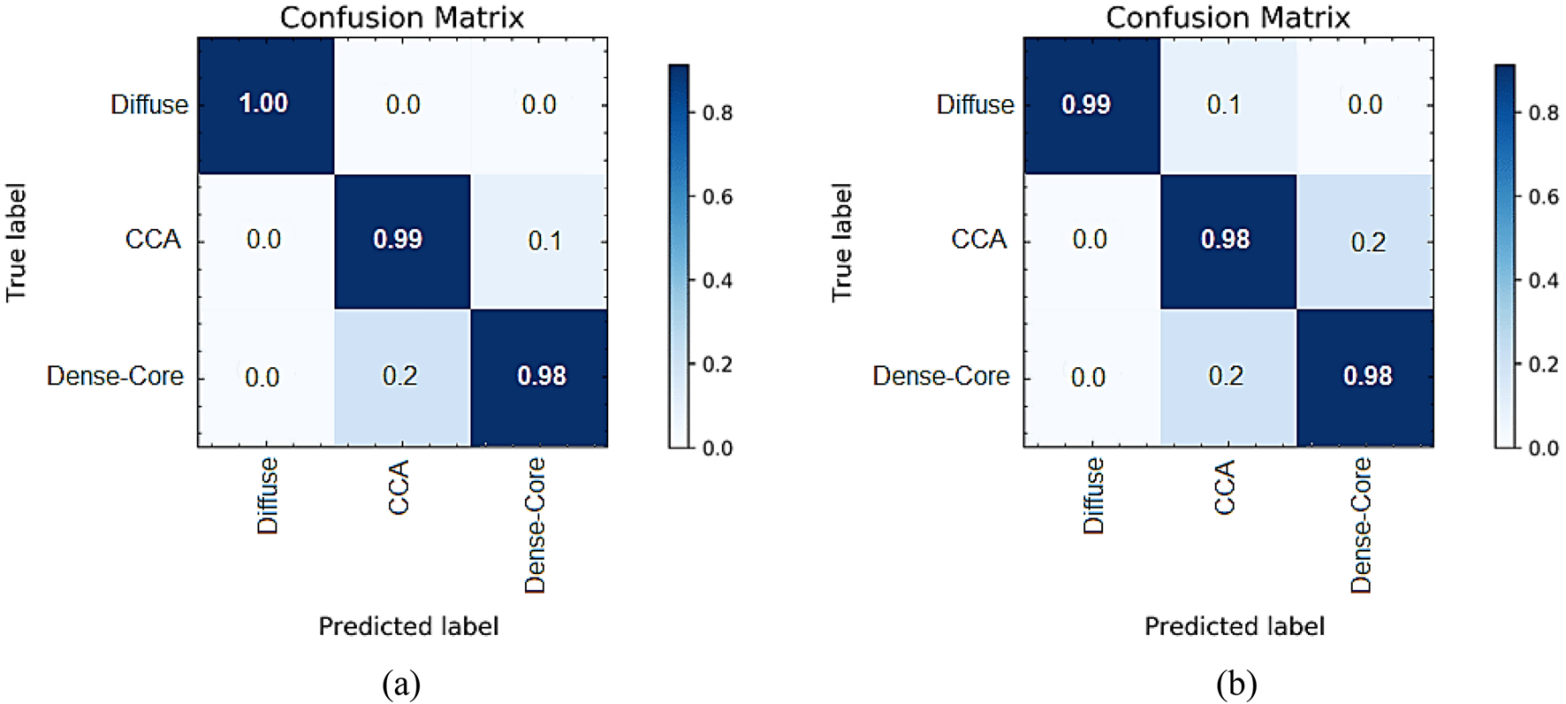
The confusion matrices analyses for (**a**) Naïve Bayes (**b**) K-Nearest Neighbor.

To ensure the effectiveness of the proposed NB algorithm using the most important features, another classifier as K-Nearest Neighbor (KNN) classifier has been used as a benchmark analysis. Several performance measures have been calculated as shown in Tables 4 and 5, and Fig. 16.

**Table 4:** The classification data.

Items	Naïve Bayes			K-Nearest Neighbor			Total
	Diffuse	CCA	Dense-core	Diffuse	CCA	Dense-core	
Subjects	100	100	100	100	100	100	300
Correctly classified images	100	99	99	99	98	98	
False classified images	0	1	2	1	2	2	
Classification *accuracy*	$\frac{297}{300}\times 100=99\%$			$\frac{295}{300}\times 100=98.3\%$

**Table 5:** Performance measure parameters for the classifiers.

Performance measures	Naïve Bayes	K-nearest neighbor
Sensitivity (Recall)$=\frac{{\varvec{T}}{\varvec{P}}}{{\varvec{T}}{\varvec{P}}+{\varvec{F}}{\varvec{N}}}\times 100$	**100**	100
Specificity $=\frac{{\varvec{T}}{\varvec{N}}}{{\varvec{T}}{\varvec{N}}+{\varvec{F}}{\varvec{P}}}\times 100$	**98.5**	98
Precision $=\frac{{\varvec{T}}{\varvec{P}}}{{\varvec{T}}{\varvec{P}}+{\varvec{F}}{\varvec{P}}}\times 100$	**97.1**	95.12
F-score $=2\times \frac{{\varvec{P}}{\varvec{r}}{\varvec{e}}{\varvec{c}}{\varvec{i}}{\varvec{s}}{\varvec{i}}{\varvec{o}}{\varvec{n}}\boldsymbol{ }\times {\varvec{R}}{\varvec{e}}{\varvec{c}}{\varvec{a}}{\varvec{l}}{\varvec{l}}}{{\varvec{P}}{\varvec{r}}{\varvec{e}}{\varvec{c}}{\varvec{i}}{\varvec{s}}{\varvec{i}}{\varvec{o}}{\varvec{n}}+{\varvec{R}}{\varvec{e}}{\varvec{c}}{\varvec{a}}{\varvec{l}}{\varvec{l}}}$	**98.5**	97.5

Significant are in value [bold].

Where *TP* true positive, *TN* true negative, *FN* false negative, and *FP* false positive.

The statistical characteristics obtained from the shown tables demonstrate that the proposed Naïve Bayes classifier has achieved the best performance. It has an accuracy of 99%. The classification method achieves a sensitivity of 100%, specificity of 98.5%, precision of 97.1%, and F-score of 98.5%.

## A Comparative analysis

A comparison of the suggested classification system with different classification parameters has been included in Table 6 to confirm its efficacy. Only one scientific paper^43^ used the same working datasets; the comparison with other researchers who used other datasets may not be fair for all algorithms. Therefore, the comparative results are as follows:

**Table 6:** Comparative evaluation.

Reference	Method	AD stages	Accuracy (%)	Sensitivity (%)	Precision (%)	Specificity (%)	datasets
Z. Tang et. al. ^43^	CNN	Diffuse, Dense-core, and CCA	98.1	–	–	–	61,370 images
**The proposed algorithm**	**Multifractal and RF**	**Diffuse, Dense-core, and CCA**	**99**	**100**	**98.5**	**97.1**	**1200 images**

Significant are in value [bold].

As shown in Table 6, the proposed methodology has achieved high accuracy with less dataset images.

## Conclusion

Alzheimer's disease (AD) is one of the most dreadful and generic classes of dementia, which causes a progressive loss of memory and cognitive function, leading to poor quality of life. The deposition of amyloid plaques is the cause of AD. Amyloid plaque aggregates are composed of amyloid-beta (Aβ), which causes the progression of AD disease. The current study proposed the assessment of the amyloid-beta using multifractal geometry. To automate the classification of AD stages, Naïve Bayes and Random Forest as a Feature selection were used. The proposed methodology achieved an accuracy of 99% and a sensitivity of 100%. The quality of the dataset images is the main limitation of the proposed methodology. It should be not less than 35% to obtain good extracted features.

### Future work

- Design a new Graphical User Interface application (GUI) to extract the most important features related to amyloid plaque morphologies as an aiding diagnosis tool.
- Using multifractal geometry as an analysis tool for detecting or classifying brain tumors.

## Data Availability

The datasets were collected from https://www.keiserlab.org/resources/.
